# Environmental Inequality in Exposures to Airborne Particulate Matter Components in the United States

**DOI:** 10.1289/ehp.1205201

**Published:** 2012-08-10

**Authors:** Michelle L. Bell, Keita Ebisu

**Affiliations:** School of Forestry and Environmental Studies, Yale University, New Haven, Connecticut, USA

**Keywords:** air pollution, chemical components, environmental justice, particulate matter, PM_2.5_, race, socioeconomic status

## Abstract

Background: Growing evidence indicates that toxicity of fine particulate matter ≤ 2.5 μm in diameter (PM_2.5_) differs by chemical component. Exposure to components may differ by population.

Objectives: We investigated whether exposures to PM_2.5_ components differ by race/ethnicity, age, and socioeconomic status (SES).

Methods: Long-term exposures (2000 through 2006) were estimated for 215 U.S. census tracts for PM_2.5_ and for 14 PM_2.5_ components. Population-weighted exposures were combined to generate overall estimated exposures by race/ethnicity, education, poverty status, employment, age, and earnings. We compared population characteristics for tracts with and without PM_2.5_ component monitors.

Results: Larger disparities in estimated exposures were observed for components than for PM_2.5_ total mass. For race/ethnicity, whites generally had the lowest exposures. Non-Hispanic blacks had higher exposures than did whites for 13 of the 14 components. Hispanics generally had the highest exposures (e.g., 152% higher than whites for chlorine, 94% higher for aluminum). Young persons (0–19 years of age) had levels as high as or higher than other ages for all exposures except sulfate. Persons with lower SES had higher estimated exposures, with some exceptions. For example, a 10% increase in the proportion unemployed was associated with a 20.0% increase in vanadium and an 18.3% increase in elemental carbon. Census tracts with monitors had more non-Hispanic blacks, lower education and earnings, and higher unemployment and poverty than did tracts without monitors.

Conclusions: Exposures to PM_2.5_ components differed by race/ethnicity, age, and SES. If some components are more toxic than others, certain populations are likely to suffer higher health burdens. Demographics differed between populations covered and not covered by monitors.

Concepts of environmental inequality and environmental justice refer to larger health burdens from environmental stressors for some populations than for others. The U.S. Environmental Protection Agency (EPA) uses “environmental justice” to refer to “fair treatment and meaningful involvement of all people regardless of race, color, national origin, or income with respect to the development, implementation, and enforcement of environmental laws, regulations, and policies,” (U.S. EPA 2010), and notes that such conditions reflect not only adverse consequences but also a lack of positive environmental, health, economic, or social benefits (U.S. EPA 2011b). The earliest studies of environmental justice focused on proximity to potentially harmful locations (e.g., incinerators) (Chavis and Lee 1987; U.S. General Accounting Office 1983).

In addition to more recent studies on proximity (Chakraborty et al. 2011; Maantay 2007; Maantay et al. 2009; Mohai and Saha 2007; Pastor et al. 2004), many other types of environmental justice issues have been researched (American Lung Association 2001; Brown 1995; Mohai et al. 2009; Waller et al. 1999). Procedural inequities could affect remediation of hazardous sites regarding priority for cleanup, time from identification of hazards to remediation, and degree of remediation or for regulatory actions, such as industry fines (Carruthers 2007; Lavelle and Coyle 1992). Adverse health outcomes may be used as a marker for environmental justice concerns, such as blood lead levels (Peters et al. 2011), which are higher for non-Hispanic black children than for non-Hispanic white children (Centers for Disease Control and Prevention 2005) or asthma, which in 1995 had a prevalence of 67.4 per 1,000 persons for African Americans and 56.2 per 1,000 persons for whites (National Institutes of Health 1999). Some populations may have a different health response to environmental conditions, meaning that a given level of exposure could have a larger impact on some groups than on others (Bell and Dominici 2008; Grineski et al. 2010; Zanobetti and Schwartz 2000). This effect modification could be related to genetics, baseline health status, access to health care, psychosocial hazards, or other factors (Bell et al. 2002; Clougherty 2010; Clougherty and Kubzansky 2009; Cory-Slechta et al. 2010; Couch and Coles 2011; Gee and Payne-Sturges 2004; Glass et al. 2009; McEwen and Tucker 2011; O’Neill et al. 2003; Ren et al. 2010; Samet and White, 2004; Son et al. 2012; Zanobetti et al. 2000).

Another type of environmental justice is whether some populations face higher exposures to contaminants than do other populations. In this article, we examine this type of environmental justice concern with respect to chemical components of airborne particulate matter (PM) with aerodynamic diameter ≤ 2.5μm (PM_2.5_). PM_2.5_ is associated with numerous adverse human health effects, especially cardiopulmonary responses (Pope and Dockery 2006). The majority of health studies on particles have estimated the effects of total PM_2.5_ mass without regard to chemical composition. In addition, the standard set by the U.S. EPA for particles is based on total mass. However, chemical structure varies widely, such as larger contributions to PM_2.5_ of nitrate in the western United States and sulfate in the eastern United States (Bell et al. 2007). Growing scientific evidence indicates that some PM_2.5_ components or sources are more harmful than others (e.g., Ito et al. 2011; Lippmann et al. 2006; Ostro et al. 2007, 2008; Peng et al. 2009). The true toxicity of different parts of the particulate mixture is unknown but is a critical research need (Health Effects Institute 2002; National Research Council 2004).

In a recent study, Miranda et al. (2011) reported that non-Hispanic blacks and persons > 64 years of age had higher PM_2.5_ exposures than did other U.S. population subgroups. Because the chemical structure of particles is likely to affect its toxicity, we investigated exposures to selected PM_2.5_ chemical components based on the hypothesis that exposures would differ by race/ethnicity, age, and socioeconomic indicators and that differences in exposures to PM_2.5_ components would be larger than differences in exposure to PM_2.5_ total mass.

## Methods

We estimated population-level exposures for different groups (e.g., race/ethnicity) to PM_2.5_ and for the following 14 PM_2.5_ components measured by the U.S. EPA’s national monitoring network: sulfate (SO_4_^2–^), nitrate (NO_3_^–^), ammonium (NH_4_^+^), organic carbon matter (OCM), elemental carbon (EC), sodium ion (Na^+^), aluminum (Al), calcium (Ca), chlorine (Cl), nickel (Ni), silicon (Si), titanium (Ti), vanadium (V), and zinc (Zn). These components were selected because they contribute ≥ 1% to total PM_2.5_ mass for yearly or seasonal averages, and/or have been associated with adverse health outcomes in previous studies including mortality, heart rate, heart rate variability, and low birth weight (Bell et al. 2007, 2009; Dominici et al. 2007; Franklin et al. 2008; Huang et al. 2012; Lippmann et al. 2006; Ostro et al. 2007, 2008; Rohr et al. 2011; Wilhelm et al. 2012).

Daily air pollution measures were obtained for 2000 through 2006 (U.S. EPA 2011a). Pollutant monitors were matched to U.S. census tracts, which are geographic units representing small subdivisions of a county and are the smallest spatial unit for which demographic variables of interest were available. Tracts from the 2000 Census (U.S. Census Bureau 2007) were designed to have an optimal population of 4,000 persons (range, 1,500–8,000) and to follow government boundaries (e.g., county), geographic features (e.g., rivers), or other identifiable features (e.g., roadways), where possible. The median land area of the 2000 census tracts in the continental United States was 5.06 km^2^.

Census tracts in the continental United States were included in our analysis if they had PM_2.5_ component monitors in operation for ≥ 3 years with ≥ 180 days of observations during the study period. Results were based on 219 monitors in 215 census tracts. Land use near monitors was 43% residential, 34% commercial, 8% industrial, 8% agricultural, and 4% forest.

We calculated long-term averages for each pollutant and 2000 census tract with a monitor for that pollutant. If multiple monitors were present for the same pollutant in a single tract, we averaged daily monitor values within a tract, and then averaged daily values to generate long-term averages. The population and area of census tracts varied. The mean (± SD) distance between a census tract’s centroid and monitor was 2.3 km ± 4.9 km (median 0.8 km; maximum 46.7 km).

For each census tract, we considered population characteristics (U.S. Census 2007):

Race: population self-identified as non-Hispanic white, non-Hispanic black or African American, non-Hispanic Asian, Hispanic, or other [SF1.P08 (Summary File 1, Table P8)]
Educational attainment: persons ≥ 25 years of age with less than a high school degree or equivalent, high school degree or equivalent, or some college (SF3.P37)
Poverty: persons in poverty using Census-defined poverty levels (SF3.P87)
Unemployment: persons ≥ 16 years of age who were unemployed, employed, or not job seekers (SF3.P43)
Age: 0–19, 20–64, or ≥ 65 years of age (SF1.P12)
Earnings: average annual earnings of those ≥ 16 years of age with earnings (SF3.P84)
Total population: (SF1.P08).

We excluded census tracts with populations ≤ 100 (*n* = 1; for tract with population = 1). For each population characteristic and category (e.g., race/ethnicity, Hispanic), we estimated the average exposure to each pollutant for that group in the United States as a whole by weighting levels in each census tract by the population as


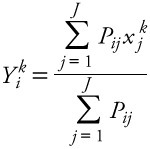
[1]

where *Y_i_^k^* is the national average estimated exposure to pollutant *k* for persons with characteristic *i* (e.g., Hispanic), *j* is the number of census tracts with pollutant data (*J =* 215), *P_i,j_* is the number of persons with characteristic *i* in census tract *j*, and *x_j_^k^* is the concentration of pollutant *k* for census tract *j*. This provides an estimate of average exposure for each pollutant and population group, accounting for population size and pollutant levels in each census tract. In addition, we performed univariate regression to estimate differences in exposure to PM_2.5_ and for each component according to census tract characteristics (e.g., percentage of persons unemployed), which are expressed as the percent change in exposure compared with overall mean levels associated with a 10% increase in a given population characteristic.

Whereas the regression analysis investigated whether some groups had higher exposures than others among areas with monitors, we further contrasted population characteristics between census tracts with and without monitors for PM_2.5_ or its components. We calculated population characteristics for census tracts with and without monitors and performed univariate logistic regression to estimate the percent increase in the probability of a census tract having a monitor with a 10% increase in each population characteristic. This analysis investigated whether some populations are better covered by the existing monitoring network than others.

## Results

**Figure 1:**
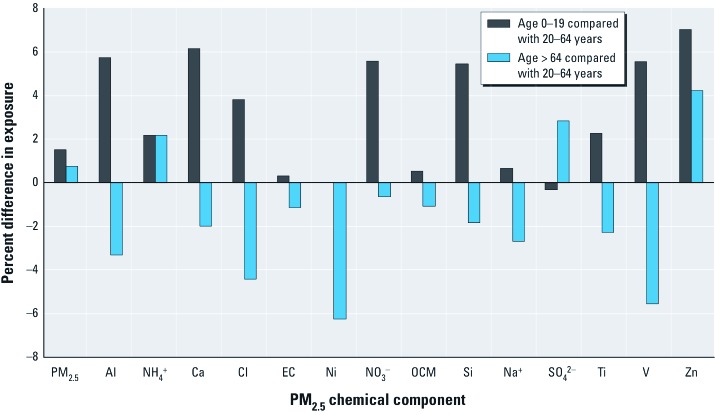
Percentage differences in exposure by age, comparing persons 0–19 or > 64 years of age with those 20–64 years of age.

Exposures among children and young adults (0–19 years of age) were as high or higher than exposures among other age groups for PM_2.5_ and all components except SO_4_^2–^, which was highest among adults ≥ 65 years of age [relative differences in exposures are presented in Figure 1; see also Supplemental Material, Table S1 (http://dx.doi.org/10.1289/ehp.1205201) for average exposure estimates according to age]. For example, those < 20 years of age had levels 7.0% higher than adults (20–64 years of age) for Zn and 6.2% higher for Ca. Older persons (≥ 65 years of age) had lower exposures than other adults (20–64 years of age) for most pollutants, with the exception of similar levels of PM_2.5_ (< 1% differences) and higher estimated exposures to NH_4_^+^, SO_4_^2–^, and Zn.

**Figure 2:**
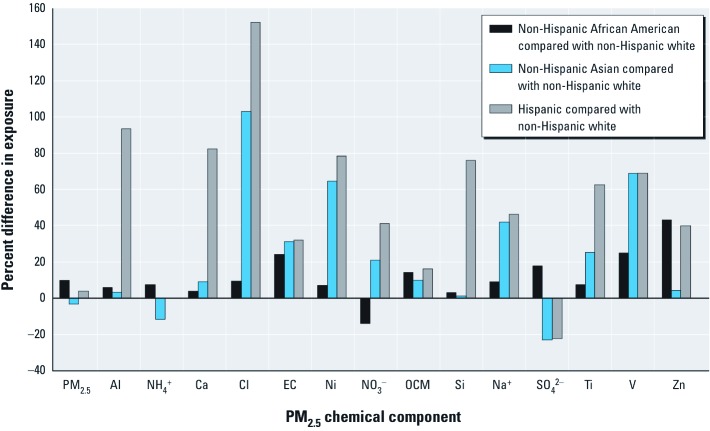
Percentage differences in exposure by race/ethnicity category, comparing non-Hispanic African American and non-Hispanic Asian to non-Hispanic white.

Non-Hispanic whites had the lowest estimated exposures for 11 of the 14 components [relative differences in exposures are presented in Figure 2; see also Supplemental Material, Table S2 (http://dx.doi.org/10.1289/ehp.1205201) for average exposure estimates according to race/ethnicity]. Hispanics had the highest estimated exposures for 10 of the 14 components and were tied with African Americans for the highest estimated exposure to V. Levels for Hispanics were higher than for non-Hispanic whites for 12 of the 14 components (e.g., 152% higher for Cl and 94% for Al). SO_4_^2–^ levels for Hispanics were 22% lower than for non-Hispanic whites. Estimated exposures were higher for African Americans than for whites for 13 of the 14 components (e.g., 43% higher for Zn, 25% for V). African Americans had the highest average exposure levels for NH_4_^+^, SO_4_^2–^, and Zn and the lowest estimated exposure to NO_3_^–^. Asians had higher estimated exposures than whites for most of the components considered (e.g., 103% for Cl, 69% for V, 64% for Ni), but they had the lowest estimated exposures of any race/ethnicity group for PM_2.5_, NH_4_^+^, and SO_4_^2–^.

**Figure 3:**
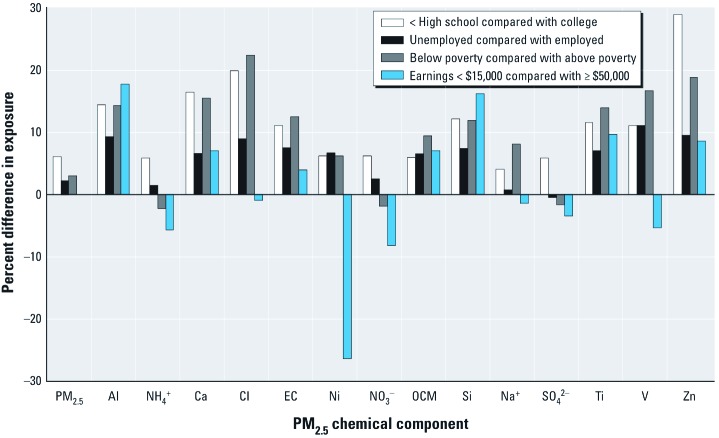
Percentage differences in exposure by category of socioeconomic indicators (education, unemployment, poverty, earnings).

In general, persons with lower-socioeconomic status (SES) had higher estimated exposures, based on indicators of education, unemployment, poverty, and earnings [relative differences in exposures are presented in Figure 3; see also Supplemental Material, Tables S3 and S4 (http://dx.doi.org/10.1289/ehp.1205201) for average exposure estimates according to the SES indicator]. Persons with less than a high school education had higher estimated exposures to PM_2.5_ and all components than did those with a college education (e.g., 6.2% higher PM_2.5_, 29% higher Zn, 20% higher Cl), and higher estimated exposures than those with a high school degree for PM_2.5_ and all components except SO_4_^2–^. Estimated exposures were ≥ 10% higher among persons without a high school education than among those with a college education for Al, Ca, Cl, EC, Si, Ti, V, and Zn.

PM_2.5_ exposures for unemployed persons were 2.3% higher than for employed persons [Figure 3 and Supplemental Material, Table S3 (http://dx.doi.org/10.1289/ehp.1205201)]. The unemployed had higher levels than employed persons for 13 of the 14 components (e.g., 11% higher for V, 9.5% for Zn). Persons in the poverty category had exposures 3.0% higher than those above the poverty line for PM_2.5_, and higher exposures for 11 of the 14 components, at ≥ 10% for Al, Ca, Cl, EC, Si, Ti, V, and Zn. Persons in the lowest earnings category had 18% higher Al and 16% higher Si exposures than did those in the highest earnings category but 26% lower levels of Ni.

Table 1 shows estimated percent differences from overall mean census-tract exposure levels with a 10% increase in individual population characteristics. For example, a 10% increase in the proportion of the Asian population was associated with 53.5% higher levels for Cl, 50.0% for V, and 45.0% for Ni. Census tracts with a higher percentage of Asians also had higher levels of EC and NO_3_^–^ and lower levels of SO_4_^2–^. A 10% increase in the proportion of Hispanics was associated with significantly higher levels of 11 of the components and lower levels of SO_4_^2–^. For example, an additional 10% of the Hispanic population was associated with increases of 18.2%, 25.4%, and 21.3% in Al, Cl, and Ni, respectively. Increases in age, unemployment, education, poverty, and earnings at the census tract level also were associated with differences in exposures. For example, a 10% increase in the proportion of the population without a high school degree was associated with increases of 19.1% in Zn and 12.2% in V.

**Table 1:** Percent increase in long-term average census tract exposure per an additional 10% increase in the population with that characteristic.

Population	PM2.5	Al	NH4+	Ca	Cl	EC	Ni	NO3–	OCM	Si	Na+	SO42–	Ti	V	Zn
Age (years)
< 20	2.69	16.0*	6.47	19.0*	–0.82	–1.37	7.50	17.7*	–0.84	12.3*	0.28	–1.46	7.27	5.00	19.5
20–64	–5.00*	–11.2	–11.3*	–14.7*	7.89	3.69	14.4	–14.2*	3.32	–7.48	6.54	–6.37	–3.18	15.0	–27.2*
≥ 65	3.07	–11.9	6.24	–11.8	–11.3	–3.41	–38.1*	–10.2	–3.81	–10.9	–11.1	13.1*	–8.64	–33.9*	7.47
Race/ethnicity
White	–1.37*	–4.93*	–1.02	–5.61*	–8.13*	–5.35*	–10.0*	–2.20	–2.72*	–4.08*	–4.01*	–0.27	–4.77*	–8.89*	–6.71*
African American	1.88*	–0.39	1.95*	0.32	–2.08	2.93*	2.50	–2.92	1.89*	–0.64	0.46	4.22*	0.23	2.22	6.10
Asian	–2.92	–5.76	–8.40	4.97	53.5*	20.3*	45.0*	19.8*	4.69	–5.47	29.9*	–18.3*	16.8*	50.0*	–5.41
Hispanic	0.13	18.2*	–0.18	16.7*	25.4*	7.17*	21.3*	12.9*	3.04*	15.9*	8.47*	–7.39*	13.2*	16.7*	4.73
Other	–16.4*	8.33	–36.5*	31.0	84.2*	23.3*	72.5*	18.4	16.8	10.8	23.3	–59.4*	16.8	52.2*	63.4
Education
< High school	4.69*	8.30*	5.26*	13.3*	11.5	9.55*	7.50	5.66	5.14*	8.04*	3.64	4.31*	9.77*	12.2*	19.1*
High school	2.34	–11.1*	7.34*	–15.7*	–19.8*	–5.38	–26.9*	–7.43	–4.66	–10.4*	–12.8*	12.5*	–13.9*	–20.6*	5.07
College	–3.55*	–2.36	–5.21*	–4.26	–2.08	–4.62*	2.50	–1.63	–2.03	–2.36	1.03	–5.96*	–2.50	–2.22	–13.4*
Unemployed	4.63	13.5	3.10	13.9	22.4	18.3*	16.9	10.3	12.0*	12.6	3.72	–2.52	15.2*	20.0*	22.0
Poverty	2.16*	7.31*	–0.69	9.89*	9.39	8.78*	11.3	–2.14	7.15*	6.19*	3.24	0.17	7.95*	8.89*	11.2
Earnings ($US/year)
< $15,000	0.07	10.4*	–3.73	10.1*	3.63	3.12	–8.13	–2.45	5.30*	10.4*	0.09	–3.77	8.64*	–1.67	5.14
$15,000–$29,999	2.61	–3.37	4.59	–6.61	–8.65	3.81	8.13	–2.74	1.24	–3.82	–1.30	7.15*	–2.50	–4.44	6.99
$30,000–$49,999	–1.55	–17.2*	4.49	–18.8*	–8.4	–10.5*	–15.0	2.43	–11.7*	–17.0*	–4.05	5.04	–18.0*	–7.78	–7.88
≥ $50,000	–1.21	–10.8	2.38	–6.79	3.25	–3.75	20.6	6.19	–6.07	–10.8	3.15	0.21	–7.05	11.1	–12.2
This table provides the percent increase in exposure level, evaluated at the mean for a 10% increase in population characteristic of a census tract. White, African American, and Asian refer to non-Hispanics. *p < 0.05.																

Supplemental Material, Table S5 (http://dx.doi.org/10.1289/ehp.1205201) compares populations of the 215 census tracts with monitors used in this study and the 64,413 tracts without monitors. In addition, 286 tracts have component monitors that did meet our inclusion criteria (e.g., sampling duration). Tracts with monitors for components had higher percentages of non-Hispanic blacks (20.5%) than did tracts without monitors (13.5%). The tracts with monitors versus those without monitors had lower SES based on education (25.5% with < high school education vs. 20.8% and 44.3% with college vs. 50.1%), unemployment (8.64% vs. 6.47%), poverty (19.9% vs. 13.4%), and earnings (39.6% for < $50,000/year vs. 33.9%). Results from univariate logistic regression indicate that a 10% increase in the population that is non-Hispanic black is associated with a 10.3% increase in the probability that a census tract has a monitor [see Supplemental Material, Table S5 (http://dx.doi.org/10.1289/ehp.1205201)]. The same increase in the population (10%) for those who had less than a high school education, who were unemployed, who were in poverty, or who had earnings < $15,000/year was associated with a 22.6%, 41.2%, 39.2%, and 37.6%, respectively, increase in the probability of a census tract having a monitor.

## Discussion

To our knowledge, this is the first study of how exposures to PM_2.5_ components may differ by population for race/ethnicity, age, and SES. In an earlier study, Marshall (2008) examined PM_2.5_ from diesel sources and hexavalent chromium based on individual-level exposure estimates in California and found higher exposures for persons who were younger (< 7 years vs. > 80 years of age), less educated (< high school vs. college), or nonwhite. Previous studies compared exposure levels of various populations for other pollutants, including PM_2.5_. U.S. counties in the lowest quantile of air quality had a higher fraction of non-Hispanic blacks and persons in poverty than did counties in the highest quantile of air quality for PM_2.5_ and ozone (Miranda et al. 2011). In the same study, the investigators found that 20% of the counties with the worst air quality for PM_2.5_ and for ozone had more persons > 64 years of age and more children < 5 years of age, respectively. Areas with parks or adjacent to parks in Los Angeles, California, had higher NO_2_ and PM_2.5_ levels in low SES or high minority neighborhoods (Su et al. 2011). In the United States, Hispanic, African-American, or Asian/Pacific Islander women had higher air pollution exposures during pregnancy than did white women after adjusting for education and other factors, based on an air pollution index that incorporated levels of PM with aerodynamic diameter ≤ 10 μm (PM_10_), ozone, carbon monoxide, NO_2_, and sulfur dioxide (Woodruff et al. 2003). In that study, Woodruff et al. (2003) found that lower education was associated with higher pollution levels, after adjustment for race/ethnicity. In Hamilton, Ontario, Canada, levels of total suspended particles (TSP) were higher in census tracts with more Latin Americans or fewer Asian Canadians, with no observable trends between TSP and black Canadians, after adjusting for SES (Buzzelli and Jerrett, 2004). In the same area, Jerrett et al. (2001) observed that TSP levels were higher in census tracts with higher dwelling values and lower income.

In Tampa, Florida, blacks, Hispanics, and persons in poverty resided in neighborhoods closer to toxic release inventory (TRI) sites, whereas whites lived closer to air pollutant monitors (Stuart et al. 2009). In California, census tracts within a mile of TRI facilities had higher fractions of minorities, especially Latinos, lower rates of home ownership, and lower incomes (Pastor et al. 2004). In Orange County, Florida, Chakraborty and Zandbergen (2007) reported that Hispanic or black children were more likely to live or attend school near TRI sources than were white children. In regions of West Virginia, Louisiana, and Maryland, African Americans lived closer to TRI sites than did whites (Perlin et al. 2001).

Our estimates are consistent with these overall trends, indicating the highest PM_2.5_ exposures for non-Hispanic blacks, the least educated, the unemployed, and those in poverty. However, overall differences were small in magnitude, with the largest difference at 9.9% higher for non-Hispanic blacks than for whites. We estimated larger disparities for exposures to PM_2.5_ components than to PM_2.5_. Whereas PM_2.5_ levels for those without a high school education were 6.2% higher than those with college, Zn levels were 29% higher. Unemployed persons had 2.3% higher PM_2.5_ than employed persons, but 11% higher levels for V. Similarly, estimated differences among race/ethnicity, earnings, or age categories were larger for many components than for PM_2.5_. The directions of the associations were different among components. For example, those in the lowest earnings category (< $15,000/year) had higher levels than those earning ≥ $50,000/year for seven components (18% higher for Al) and lower levels for seven components (26% lower for Ni).

We used community-level exposures for census tracts. More precise measures would incorporate spatial heterogeneity (Peng and Bell 2010), as well as daily activity patterns, indoor exposures (e.g., environmental tobacco smoke), inhalation rates, and occupational exposures at the individual level. Many of these factors (e.g., occupation) may differ by population. Exposures were estimated from ambient monitors, and thus do not reflect the personal exposures of all individuals within the census tract.

Our research does not disentangle demographic characteristics of race/ethnicity, education, unemployment, poverty, and earnings; and many population characteristics co-vary [see Supplemental Material, Table S6 (http://dx.doi.org/10.1289/ehp.1205201) for correlations]. For example, race, education, earnings, and poverty were correlated. Future work could examine patterns in population characteristics in relation to PM_2.5_ component exposures and patterns related to community factors, such as urbanicity and property values.

Only 215 census tracts had PM_2.5_ component monitors meeting the inclusion criteria, covering 0.3% of the population. The monitor coverage hinders ability to fully investigate equity issues, especially for rural populations, which likely have different characteristics. As population demographics and chemical composition of particles differ dramatically by region (Bell et al. 2007), the geographical distribution of monitors could affect results. In this study, 37% of monitors were in the South (defined by U.S. Census regions), 27% in the Midwest, 19% in the West, and 17% in the Northeast. Future research may consider alternative methods of estimating exposure, such as air quality modeling and satellite imagery (Anderson et al. 2012; Bell 2006; Boldo et al. 2011; Fann et al. 2012), to estimate exposures for a larger population.

Our results show that populations potentially at risk for higher exposures to components do not appear to be underrepresented in areas with monitors compared with areas without monitors. This contrasts with the study by Miranda et al. (2011) that found that U.S. counties without sufficient monitoring for PM_2.5_ and ozone had fewer non-Hispanic blacks, Hispanics, and persons < 5 years of age and a higher percentage of persons > 64 years of age. Our findings may differ because of the use of census tracts (median land area = 5.06 km^2^; SD = 571 km^2^) rather than counties (median land area = 1,582 km^2^; SD = 3,375 km^2^) and because of differences between monitoring networks for PM_2.5_ and PM_2.5_ components. Other studies also have shown links between population characteristics and monitoring networks. In São Paulo, Brazil, areas with higher SES were more likely to have PM_10_ and ozone monitors (Bravo and Bell 2010).

Additional challenges in this area of research include the choice and interpretation of SES indicators, because true SES relates to historical conditions, full sources of income, as well as access to resources beyond official earnings, neighborhood-level SES, insurance, access to health care, use of health care systems, and social networks (Bell et al. 2002; O’Neill et al. 2003). The interpretation of SES indicators can vary by region or subculture. Subjective measures of SES include factors such as satisfaction with position, comparison to peers, and perception of financial security. Perceived and actual SES may differ and can have different trends by population (Brown et al. 2008). Traditional measures of SES (e.g., income, education) can be supplemented with subjective social status measures, which in some cases may be more closely linked to health outcomes than to conventional measures (Dennis et al. 2012; Singh-Manoux et al. 2003).

The 14 PM_2.5_ chemical components investigated here were selected because they contribute ≥ 1% to PM_2.5_ total mass and/or were found to be potentially harmful to health in earlier studies. However, the full health impacts of various particle mixtures and the identities of the most harmful components or set of components are unknown. A further complication is that all components come from multiple sources, although some components are more strongly linked to some sources than to others (e.g., Ni and V from oil combustion, SO_4_^2–^ from coal combustion, Si from road dust).

A growing body of scientific literature, including epidemiological and toxicological studies, indicates health associations with various PM_2.5_ chemical components (U.S. EPA 2009). For example, results of toxicological studies using animal models and human-cell cultures suggest the possibility of adverse respiratory effects for Zn (Gerlofs-Nijland et al. 2007; Wu et al. 2003, 2004), Al (Graff et al. 2007), V (Veranth et al. 2007), SO_4_^2–^ (Riley et al. 2005), and NO_3_^–^ (Huang et al. 2003). Animal models have shown associations with cardiovascular outcomes, such as for zinc (Bagaté et al. 2004, 2006). As additional information becomes available on which chemical components and related sources are most harmful, future studies could examine how such exposures differ by population.

## Conclusions

Our estimates suggest differences among populations in PM_2.5_ component exposures. However, exposure differences may only partly determine whether health impacts from these pollutants are greater in some population groups than in others. The actual difference among groups for health burdens from PM_2.5_ or its components depends not only on the distribution of exposure, but whether effects are modified by population characteristics. In other words, although we show in this study that some populations have higher exposures than others, a separate issue is whether a given exposure results in the same health response across populations. Methods for risk assessment are needed to assess different effects of environmental exposures across populations and communities that incorporate temporal and spatial connections among risk factors in real-world settings (Schwartz et al. 2011).

Our findings highlight the need for additional research to understand health responses to complex pollutant mixtures, as opposed to effects of individual pollutants. Advances in this field of research are further complicated by inadequate data on multiple pollutants and limitations in statistical methods and exposure assessment (Dominici et al. 2010). However, our work takes a step toward that goal by providing information on differences in exposures that can be used to inform future studies investigating differential health impacts from PM_2.5_ components and the particulate mixture.

## Supplemental Material

(37 KB) PDF
