# Supplementary material for: Environmental Inequality in Exposures to Airborne Particulate Matter Components in the United States
Source: Environ Health Perspect. 2012 Aug 10;120(12):1699–704. doi: 10.1289/ehp.1205201 (PMC3546368; doi:10.1289/ehp.1205201)
Supplement: (37 KB) PDF [file ehp.1205201.s001.pdf]

Supplemental Material

Environmental Inequality in Exposures to Airborne Particulate Matter Components in the United States

Michelle L. Bell and Keita Ebisu

Table of Contents page

Supplemental Material Table S1. Average (± standard deviation) estimated exposure to $PM_{2.5}$ total mass and $PM_{2.5}$ chemical components in the U.S., overall and by age [μg/m^3] 2

Supplemental Material Table S2. Average (± standard deviation) estimated exposure to $PM_{2.5}$ total mass and $PM_{2.5}$ chemical components in the U.S., overall and by race/ethnicity categories [μg/m^3] 3

Supplemental Material Table S3. Long-term average (± standard deviation) estimated exposure to $PM_{2.5}$ total mass and $PM_{2.5}$ chemical components in the U.S., by educational attainment and employment status [μg/m^3] 4

Supplemental Material Table S4. Long-term average (± standard deviation) estimated exposure to $PM_{2.5}$ total mass and $PM_{2.5}$ chemical components in the U.S., by poverty status and earnings [μg/m^3] 5

Supplemental Material Table S5. Comparison of population characteristics for census tracts with and without monitors for $PM_{2.5}$ chemical components 6

Supplemental Material Table S6. Correlation of census tract population characteristics 7

Supplemental Material Table S1. Average (± standard deviation) estimated exposure to $PM_{2.5}$ total mass and $PM_{2.5}$ chemical components in the U.S., overall and by age [μg/m^3]

Population or Pollutant	*Total Population*	*Age*		
		≤ 19 yrs	*20 to 64 yrs*	*≥ 65 yrs*
Population	867,815	250,927	511,921	104,967
$PM_{2.5}$ total mass	13.4 ± 2.87	13.5 ± 2.87	13.3 ± 2.86	13.4 ± 2.90
Al	0.0335 ± 0.0291	0.0350 ± 0.0319	0.0331 ± 0.0280	0.032 ± 0.0274
NH_4^+	1.39 ± 0.545	1.41 ± 0.541	1.38 ± 0.547	1.41 ± 0.544
Ca	0.0561 ± 0.0425	0.0587 ± 0.0467	0.0553 ± 0.0407	0.0542 ± 0.0398
Cl	0.0342 ± 0.0477	0.0353 ± 0.0488	0.0340 ± 0.0472	0.0325 ± 0.0470
EC	0.618 ± 0.266	0.621 ± 0.267	0.619 ± 0.264	0.612 ± 0.268
Ni	0.0016 ± 0.0019	0.0016 ± 0.0019	0.0016 ± 0.0019	0.0015 ± 0.0017
NO_3^-	1.64 ± 1.14	1.70 ± 1.20	1.61 ± 1.11	1.60 ± 1.09
OCM	3.75 ± 1.36	3.77 ± 1.37	3.75 ± 1.35	3.71 ± 1.38
Si	0.112 ± 0.0809	0.116 ± 0.0878	0.110 ± 0.0780	0.108 ± 0.0768
Na^+	0.149 ± 0.0823	0.150 ± 0.0850	0.149 ± 0.0820	0.145 ± 0.0769
$SO_4^=$	3.18 ± 1.30	3.16 ± 1.28	3.17 ± 1.30	3.26 ± 1.32
Ti	0.0044 ± 0.0025	0.0045 ± 0.0027	0.0044 ± 0.0024	0.0043 ± 0.0024
V	0.0018 ± 0.0014	0.0019 ± 0.0014	0.0018 ± 0.0014	0.0017 ± 0.0013
Zn	0.0146 ± 0.0196	0.0152 ± 0.0214	0.0142 ± 0.0187	0.0148 ± 0.0193

Supplemental Material Table S2. Average (± standard deviation) estimated exposure to $PM_{2.5}$ total mass and $PM_{2.5}$ chemical components in the U.S., overall and by race/ethnicity categories [μg/m^3]

Population or Pollutant	*Non-Hispanic white*	*Non-Hispanic Black/African-American*	*Non-Hispanic Asian*	*Hispanic*	*Other race*
Population	555,853	155,159	26,066	108,560	22,177
$PM_{2.5}$ total mass	13.1 ± 2.81	14.4 ± 2.08	12.7 ± 3.47	13.6 ± 3.48	12.8 ± 3.26
Al	0.0296 ± 0.0190	0.0313 ± 0.0152	0.0306 ± 0.0288	0.0573 ± 0.0601	0.0341 ± 0.0259
NH_4^+	1.38 ± 0.536	1.4 ± 0.417	1.22 ± 0.656	1.38 ± 0.672	1.25 ± 0.619
Ca	0.0502 ± 0.0291	0.052 ± 0.0323	0.0547 ± 0.0401	0.0916 ± 0.0802	0.0604 ± 0.0398
Cl	0.0273 ± 0.0389	0.0299 ± 0.0442	0.0554 ± 0.052	0.0689 ± 0.0705	0.0420 ± 0.0442
EC	0.564 ± 0.246	0.700 ± 0.267	0.738 ± 0.297	0.745 ± 0.270	0.659 ± 0.253
Ni	0.0014 ± 0.0016	0.0015 ± 0.0017	0.0023 ± 0.0023	0.0025 ± 0.0029	0.0017 ± 0.0018
NO_3^-	1.58 ± 1.00	1.36 ± 0.826	1.91 ± 1.44	2.23 ± 1.71	1.73 ± 1.21
OCM	3.57 ± 1.41	4.08 ± 1.02	3.92 ± 1.24	4.15 ± 1.33	3.91 ± 1.49
Si	0.101 ± 0.0564	0.104 ± 0.0465	0.102 ± 0.0766	0.178 ± 0.159	0.114 ± 0.0746
Na^+	0.136 ± 0.0698)	0.148 ± 0.0703	0.193 ± 0.101	0.199 ± 0.119	0.159 ± 0.0884
$SO_4^=$	3.20 ± 1.34	3.77 ± 0.841	2.47 ± 1.29	2.49 ± 1.16	2.66 ± 1.36
Ti	0.0040 ± 0.0018	0.0043 ± 0.0017	0.0050 ± 0.0033	0.0065 ± 0.0044	0.0046 ± 0.0024
V	0.0016 ± 0.0011	0.0020 ± 0.0016	0.0027 ± 0.0019	0.0027 ± 0.0018	0.0020 ± 0.0015
Zn	0.0128 ± 0.0153	0.0183 ± 0.0311	0.0133 ± 0.0113	0.0179 ± 0.0164	0.0178 ± 0.0263

Supplemental Material Table S3. Long-term average (± standard deviation) estimated exposure to $PM_{2.5}$ total mass and $PM_{2.5}$ chemical components in the U.S., by educational attainment and employment status [μg/m^3]

Population or Pollutant	*Educational Attainment*			*Employment Status*		
	< High school	*High school*	*College*	*Unemployed*	*Employed*	*Non-Jobseeker*
Population	130,611	164,169	249,517	32,069	382,572	258,724
$PM_{2.5}$ total mass	13.8 ± 2.90	13.4 ± 2.77	13.0 ± 2.85	13.5 ± 2.98	13.2 ± 2.87	13.5 ± 2.84
Al	0.0365 ± 0.0343	0.0314 ± 0.0245	0.0319 ± 0.0269	0.0352 ± 0.0287	0.0322 ± 0.0272	0.0342 ± 0.0294
NH_4^+	1.43 ± 0.554	1.42 ± 0.530	1.35 ± 0.546	1.39 ± 0.564	1.37 ± 0.546	1.40 ± 0.539
Ca	0.0623 ± 0.0521	0.0521 ± 0.0359	0.0535 ± 0.0365	0.0572 ± 0.0440	0.0537 ± 0.0385	0.0574 ± 0.0439
Cl	0.0391 ± 0.0528	0.0309 ± 0.0464	0.0326 ± 0.0450	0.0355 ± 0.0478	0.0326 ± 0.0457	0.0348 ± 0.0494
EC	0.666 ± 0.283	0.608 ± 0.272	0.599 ± 0.252	0.647 ± 0.277	0.602 ± 0.259	0.633 ± 0.269
Ni	0.0017 ± 0.0020	0.0015 ± 0.0016	0.0016 ± 0.0021	0.0016 ± 0.0019	0.0015 ± 0.0019	0.0015 ± 0.0018
NO_3^-	1.70 ± 1.28	1.57 ± 1.02	1.60 ± 1.05	1.65 ± 1.21	1.61 ± 1.08	1.60 ± 1.13
OCM	3.90 ± 1.30	3.69 ± 1.38	3.68 ± 1.37	3.92 ± 1.46	3.68 ± 1.35	3.80 ± 1.34
Si	0.120 ± 0.0944	0.105 ± 0.0697	0.107 ± 0.0749	0.116 ± 0.0804	0.108 ± 0.0761	0.114 ± 0.0815
Na^+	0.154 ± 0.0894	0.142 ± 0.0760	0.148 ± 0.0793	0.148 ± 0.0828	0.147 ± 0.0806	0.149 ± 0.0816
$SO_4^=$	3.26 ± 1.24	3.32 ± 1.28	3.08 ± 1.34	3.14 ± 1.29	3.15 ± 1.32	3.24 ± 1.27
Ti	0.0048 ± 0.0029	0.0042 ± 0.0022	0.0043 ± 0.0023	0.0046 ± 0.0025	0.0043 ± 0.0023	0.0045 ± 0.0025
V	0.0020 ± 0.0015	0.0017 ± 0.0013	0.0018 ± 0.0014	0.0020 ± 0.0016	0.0018 ± 0.0014	0.0018 ± 0.0014
Zn	0.0169 ± 0.0228	0.0145 ± 0.0198	0.0131 ± 0.0157	0.0150 ± 0.0212	0.0137 ± 0.0177	0.0151 ± 0.0203

Supplemental Material Table S4. Long-term average (± standard deviation) estimated exposure to $PM_{2.5}$ total mass and $PM_{2.5}$ chemical components in the U.S., by poverty status and earnings [$\mu g/m^3$]

Population or Pollutant	*Poverty Status*		*Annual Earnings*			
	Below Poverty	*Above Poverty*	*<$15,000*	*$15,000-$29,999*	*$30,000-$49,999*	*≥$50,000*
Population	145,802	680,158	176,559	138,189	90,465	54,126
$PM_{2.5}$ total mass	13.7 ± 2.98	13.3 ± 2.86	13.2 ± 2.97	13.3 ± 2.89	13.2 ± 2.76	13.2 ± 2.60
Al	0.0374 ± 0.0331	0.0327 ± 0.0288	0.0344 ± 0.0289	0.0323 ± 0.0261	0.0310 ± 0.0258	0.0292 ± 0.0252
NH_4^+	1.37 ± 0.567	1.40 ± 0.547	1.34 ± 0.553	1.38 ± 0.550	1.39 ± 0.528	1.42 ± 0.525
Ca	0.0633 ± 0.0533	0.0548 ± 0.0403	0.0562 ± 0.0430	0.0533 ± 0.0370	0.0518 ± 0.0348	0.0525 ± 0.0342
Cl	0.0409 ± 0.0536	0.0334 ± 0.0469	0.0331 ± 0.0469	0.0323 ± 0.0452	0.0316 ± 0.0446	0.0334 ± 0.0451
EC	0.684 ± 0.282	0.608 ± 0.263	0.609 ± 0.260	0.612 ± 0.261	0.592 ± 0.253	0.595 ± 0.258
Ni	0.0017 ± 0.0019	0.0016 ± 0.0019	0.0014 ± 0.0017	0.0015 ± 0.0019	0.0015 ± 0.0018	0.0019 ± 0.0026
NO_3^-	1.62 ± 1.26	1.65 ± 1.12	1.57 ± 1.13	1.60 ± 1.09	1.62 ± 1.02	1.71 ± 0.987
OCM	4.04 ± 1.36	3.69 ± 1.36	3.79 ± 1.40	3.72 ± 1.33	3.60 ± 1.30	3.54 ± 1.24
Si	0.122 ± 0.0921	0.109 ± 0.0797	0.115 ± 0.0806	0.108 ± 0.0736	0.104 ± 0.0720	0.0989 ± 0.0696
Na^+	0.159 ± 0.0908	0.147 ± 0.0815	0.147 ± 0.0812	0.147 ± 0.0811	0.145 ± 0.0773	0.149 ± 0.0809
$SO_4^=$	3.13 ± 1.27	3.18 ± 1.31	3.09 ± 1.32	3.19 ± 1.31	3.20 ± 1.29	3.20 ± 1.26
Ti	0.0049 ± 0.0028	0.0043 ± 0.0024	0.0045 ± 0.0025	0.0043 ± 0.0023	0.0041 ± 0.0022	0.0041 ± 0.0021
V	0.0021 ± 0.0016	0.0018 ± 0.0014	0.0018 ± 0.0013	0.0018 ± 0.0014	0.0017 ± 0.0014	0.0019 ± 0.0016
Zn	0.0170 ± 0.0249	0.0143 ± 0.0188	0.0139 ± 0.0187	0.0138 ± 0.0182	0.0134 ± 0.0165	0.0128 ± 0.0128

Supplemental Material Table S5. Comparison of population characteristics for census tracts with and without monitors for $PM_{2.5}$ chemical components

	Population characteristics Mean ± standard deviation		*% increase in probability of having $PM_{2.5}$ component monitor per 10% increase in population characteristic of census tract (95% confidence interval)*
	Census tracts with monitors	*Census tracts without monitors*	
Population	867,815	279,326,737	
Number of census tracts	215	64,413	
Race			
Non-Hispanic White	63.3% ± 31.6%	68.8% ± 30.3%	-5.4 (-9.2, -1.5)
Non-Hispanic Black	20.5% ± 28.6%	13.5% ± 23.5%	10.3 (5.5, 15.4)
Non-Hispanic Asian	2.74% ± 5.65%	3.35% ± 7.16%	-14.0 (-32.3, 9.2)
Hispanic	10.8% ± 17.2%	11.5% ± 18.9%	-2.2 (-9.3, 5.4)
Other	2.66% ± 2.67%	2.77% ± 5.62%	-4.0 (-26.7, 25.7)
Age			
≤19 years	28.4% ± 7.15%	28.2% ± 6.98%	3.4 (-14.7, 25.2)
20 to 64 years	59.1% ± 6.65%	58.8% ± 6.65%	6.3 (-12.8, 29.6)
≥65 years	12.6% ± 5.22%	13.0% ± 7.08%	-9.0 (-25.8, 11.5)
Education			
< High school	25.5% ± 14.0%	20.8% ± 14.0%	22.6 (12.9, 33.1)
High school	30.3% ± 8.97%	29.1% ± 10.2%	12.5 (-1.5, 28.4)
College	44.3% ± 17.6%	50.1% ± 18.9%	-15.9 (-22.0, -9.3)
Employment (unemployed)	8.64% ± 6.17%	6.47% ± 5.84%	41.2 (24.8, 59.9)
Poverty (below poverty)	19.9% ± 14.1%	13.4% ± 11.6%	39.2 (28.6, 50.7)
Annual Earnings			
<$15,000	39.6% ± 13.2%	33.9% ± 11.7%	37.6 (26.2, 50.0)
$15,000-$29,99	30.8% ± 7.58%	28.6% ± 7.90%	44.1 (21.1, 71.5)
$30,000-$49,999	19.1% ± 6.73%	21.5% ± 6.43%	-42.7 (-53.0, -30.3)
≥$50,000	10.5% ± 8.75%	16.0% ± 12.0%	-43.9 (-52.8, -33.3)

Note: White, black, and Asian refer to non-Hispanics. Supplemental Table 1 compares census tracts with monitors that met our exclusion criteria and tracts without monitors.

Supplemental Material Table S6. Correlation of census tract population characteristics

	Race/Ethnicity					*Age (years)*			*Education*			*Employment*	*Poverty*	*Earnings*		
	White	Black	Asian	Hispanic	Other race	<20	20-64	≥65	< High school	High school	College	Unemployed	Below poverty line	≥$50,000	$30,000-$49,999	$15,000-$29,999
Annual Earnings																
<$15,000	-0.43	0.35	0.06	0.17	0.14	0.01	-0.16	-0.33	0.46	-0.15	-0.28	**0.69**	**0.79**	**-0.61**	**-0.83**	-0.30
$15,000-$29,999	-0.18	0.17	-0.11	0.08	0.02	-0.10	-0.08	0.04	0.27	0.45	-0.45	-0.16	-0.03	-0.47	0.07	
$30,000-$49,999	0.50	-0.39	-0.08	-0.22	-0.13	0.00	0.13	0.26	-0.46	0.24	0.24	**-0.60**	**-0.73**	0.41		
≥$50,000	0.42	-0.38	0.07	-0.15	-0.13	0.08	0.21	0.25	-0.57	-0.35	**0.63**	-0.45	**-0.61**			
Poverty	**-0.65**	0.56	0.04	0.21	0.18	-0.09	-0.22	-0.40	**0.63**	-0.13	-0.43	**0.66**				
Unemployed	-0.50	0.38	0.11	0.23	0.14	0.03	-0.17	-0.36	0.47	-0.13	-0.30					
Education																
College	0.40	-0.35	0.18	-0.23	0.08	0.07	0.22	0.12	**-0.86**	**-0.62**						
High school	0.19	0.02	-0.33	-0.25	-0.22	-0.16	-0.14	0.11	0.14							
< High school	**-0.63**	0.42	-0.01	0.45	0.04	0.02	-0.19	-0.22								
Age																
≥65 years	0.24	-0.23	0.01	-0.04	-0.17	0.42	0.57									
20-64 years	0.07	-0.19	0.11	0.16	-0.05	**0.76**										
<20 years	-0.08	-0.11	0.07	0.32	-0.06											
Race/Ethnicity																
Other race	-0.09	-0.10	0.30	0.09												
Hispanic	-0.44	-0.15	0.16													
Non-Hispanic Asian	-0.17	-0.13														
Non-Hispanic black	**-0.79**															

Note: Correlations are not shown for mutually exclusive binary categories (employment, poverty). For example, correlations between a population characteristics and the percentage unemployed is -1x the correlation with the percentage employed. White, black, and Asian refer to non-Hispanics. Values that are ≥0.6 or ≤-0.6 are bolded,
